# The Functional Neuroses

**Published:** 1915-08

**Authors:** 


**The Functional Neuroses,** Rung, Medical Herald Kansas City, Mo., quoting a prominent authority says, "People who constantly complain of illness die of disease at a ripe old age, their troubles have been functional." He says that fully three-fourths of human illness is of a functional nature, that there is no true pathological condition present, but that although it is functional, it is not always controlable. There is a cause, and this cause is situated in the central nervous system, the brain. He quotes, Cotton, Iowa State Medical Journal who says, "Without the nervous system there would be no pains, no aches: but without the nervous system there would be no life; for it is this system that controls and operates every organ, every function of the human body at all times."

Great stress is laid on the fact that the nervous system has a specific nourishment, the same as the blood, bone, muscles, etc. He says this is phosphorus, lecithin and nuclein, and on its presence, and its normal use depends the action of the bodily functions. As to supplying nerve nutrition, and its use by the system, he quotes a L. R. C. P. of England, who says, "The day has passed when we must guess; the phosphatometer tells us in a few minutes whether sedatives or nerve tonics are necessary,'' and on these depend the relief of practically all functional conditions; it is only a question of which to use.

Gastro-intestinal disturbances are carefully considered, he claiming that fully 90% of dyspepsias are due to neuroses, usually a want of nutrition for the nervous system: they are easily, quickly and permanently relieved by attention to this system.

Two typical cases are reported: one a dyspepsia with gas accumulations, necessitating digestive tablets for two years. An index was taken, being 90% minus, phosphorus was ordered in the shape of Co. Phos. Mixture (Dowd): relief was almost immediate remaining permanent, after the administration of this mixture for two weeks. The second case was pain in the legs, backache, dizziness, obstinate constipation with more or less digestive irritation following food.

No pathological condition was evident following careful examination: blood pressure was 160 (patient 38) and an index of 50% plus.

This case being acute, Fl. Ex. Valerian was given in connection with resin podoph. In a few days there was entire relief, blood pressure 135, and index fell to practically normal in a week, patient has been well for over a year.

It is now only a question of whether we should go on guessing, or use ten minutes and be certain.

